# Evaluating the Potential for Cross-Interactions of Antitoxins in Type II TA Systems

**DOI:** 10.3390/toxins12060422

**Published:** 2020-06-26

**Authors:** Chih-Han Tu, Michelle Holt, Shengfeng Ruan, Christina Bourne

**Affiliations:** Department of Chemistry and Biochemistry, University of Oklahoma, Norman, OK 73019, USA; chihhantu@ou.edu (C.-H.T.); mnholt1217@ou.edu (M.H.); shengfengruan@ou.edu (S.R.)

**Keywords:** cognate interactions, cross-interactions, molecular insulation, toxin, antitoxin, TA systems, addiction, anti-addiction

## Abstract

The diversity of Type-II toxin–antitoxin (TA) systems in bacterial genomes requires tightly controlled interaction specificity to ensure protection of the cell, and potentially to limit cross-talk between toxin–antitoxin pairs of the same family of TA systems. Further, there is a redundant use of toxin folds for different cellular targets and complexation with different classes of antitoxins, increasing the apparent requirement for the insulation of interactions. The presence of Type II TA systems has remained enigmatic with respect to potential benefits imparted to the host cells. In some cases, they play clear roles in survival associated with unfavorable growth conditions. More generally, they can also serve as a “cure” against acquisition of highly similar TA systems such as those found on plasmids or invading genetic elements that frequently carry virulence and resistance genes. The latter model is predicated on the ability of these highly specific cognate antitoxin–toxin interactions to form cross-reactions between chromosomal antitoxins and invading toxins. This review summarizes advances in the Type II TA system models with an emphasis on antitoxin cross-reactivity, including with invading genetic elements and cases where toxin proteins share a common fold yet interact with different families of antitoxins.

## 1. Introduction

Toxin–antitoxin (TA) systems are abundant in bacterial and archaeal chromosomes as well as extra-chromosomal genetic elements including plasmids, phages, and transposons [1,2,3,4,5,6,7]. TA systems have been the subject of numerous reviews that describe their typically bicistronic operon encoding a toxin that targets essential cellular process and the cognate neutralizing antitoxin [8,9,10,11,12,13,14,15,16]. Toxin proteins are able to manipulate their bacterial host cells in powerful ways. This drives great interest in understanding their functions and the potential to utilize them for biotechnology and bacterial control strategies [17,18,19,20,21,22,23,24,25,26].

**Figure 1:**
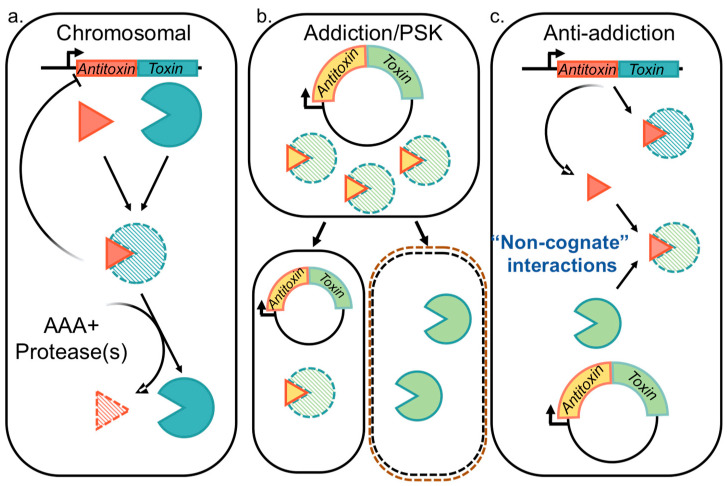
Paradigms for Type II toxin–antitoxin (TA) system functions span roles in (**a**) physiology mediated by chromosomally-encoded systems, in addition to potential roles (**b**) as addiction modules on mobile genetic elements. An alternative may arise (**c**) when multiple TA systems are present within the same cell, giving rise to an anti-addiction role. Experimental evidence has demonstrated that chromosomally-encoded systems can protect individual cells from external stressors (**a**). When TA systems are encoded on mobile genetic elements (pictured on a plasmid) they can function as “Addiction” elements that enforce retention (also referred to as post-segregation killing, or PSK) (**b**). A hybrid paradigm combines these two functionalities, wherein a chromosomally-encoded antitoxin (or toxin) can neutralize an invading toxin, thus providing an anti-addiction function (**c**).

Depending on the molecular identity of the antitoxin, as well as the mechanism by which it neutralizes the toxin, the known TA systems have been classified into six different types. While the toxin is typically a protein, the antitoxin is either a noncoding RNA (in Type I and III) or a protein (in Type II, IV, V and VI) [15,18,25,27,28,29,30]. Limited numbers of Type III TA pairs [31,32,33,34] and IV TA pairs have been identified [35,36,37]. Type V systems identified to date are the GhoST system in *Escherichia coli* K strains and the orphan OrtT toxin from *Salmonella* [38,39,40]. Similarly, the Type VI system is currently comprised of the Soc system in *Caulobacter crescentus* [41]. However, continuing surprises are challenging the canonical TA system paradigms. These include the integration of some TA systems into alarmone signaling pathways [42,43] and recognition of alternatives to protease-dependent regulation such as acetylation of antitoxins and/or chaperone protection [44,45,46], in addition to widespread variations on the bicistronic antitoxin–toxin arrangement [44,47]. In comparison, Type I and II TA systems are well-studied, and Type II systems appear in thousands of bacterial loci that are accessible and searchable through tailored web portals; to-date, the TADB webserver lists more than 6000 Type II systems with approximately 10% of these located on plasmids [1,2,7,48,49,50,51]. In Type II systems the antitoxin partner plays a dual role by neutralizing the cognate toxin and by mediating transcriptional regulation through binding to its promoter, making it the lynchpin of Type II TA system functions [52] (Figure 1). As such, many of the proposed functions of these systems are predicated on the ability of antitoxins to potentially neutralize multiple homologous toxins, as explored in the current review.

## 2. Paradigms for Type II TA Systems

Type II TA systems were originally recognized as mediators of plasmid addiction, also termed “post-segregational killing” (PSK) (Figure 1b) [53,54,55,56,57]. Subsequent genome sequencing efforts identified them throughout bacteria and archaea [1,2,7], with those presenting some levels of similarity to the plasmidic versions but not other related genomic versions referred to as “xenologs” to highlight their likelihood of arising through horizontal gene transfer [58]. Analysis of xenolog distribution led to insight that these TA systems are a form of bacterial immunity with implications for protection from invading genetic material [28,59,60,61]. Therefore, the roles of given Type II TA systems described in Figure 1 remain unsettled and are likely overlapping.

### 2.1. Functions Attributed to Chromosomal Type II TA Systems 

The prevailing evidence favors the stochastic accumulation of TA systems through horizontal gene transfer, with a predominance of phage-derived systems at “hotspots” of genetic diversity within a given bacterial species [2,7,58,62,63,64,65]. As such, their potential role has been a subject of intense debate. For many, it has been demonstrated that they are located within integrated (at least previously) mobile genetic elements, and further, some of these function as addiction modules for those integrated elements (see Section 2.2, below). Some have proposed that integration of a TA system into a bacterial chromosome has allowed it to be co-opted to maintain normal physiological homeostasis in response to environmental or other changes in growth conditions, sometimes termed “domestication” or linked to “accessory” genomic content [8,66]. Further, for some TA systems there likely exists a functional overlap of these ideas, encompassing both genetic addiction and the potential for usefulness to the cells (Figure 1) [27,67].

Some investigations have documented an attenuation of chromosomally-encoded TA system toxicity. This has arisen either through sequence changes resulting in lower affinity for interaction with the cellular target [68,69,70,71,72] or by attenuation of expression via changes in the promoter regions [73,74,75]. A loss of function, termed “degeneration”, has also been observed for some systems, such as a chromosomal CcdAB system in *E. coli* [62]. Attenuation of toxicity provides an opportunity for toxins to regulate their cellular target without killing the host cell. While this led to the long-touted idea of TA system involvement in persister cell generation, it can also be more generalized simply as the ability of cells to withstand external stressors by reducing metabolism or protecting critical cellular targets from damage [16,76,77]. 

Gyrase-targeting systems CcdAB and ParDE, each originally identified as mediators of PSK, have been documented to provide useful advantages to their host cells. The CcdB toxin, carried on the F plasmid and the chromosomal xenolog in *E. coli,* both were observed to increase cellular survival to heat, anti-gyrase compounds, and other antibiotics [68,78]. An analogous protective effect from anti-gyrase antibiotics was observed for the chromosomal ParE from *Pseudomonas aeruginosa* [71]. The protection of host cells from thermal stress has also been observed for the ParE toxin carried on the RK2 plasmid [79]. *Caulobacter crescentus* carries three functional chromosomal ParDE systems, and these were observed to offer protection to stressors, as did one of a ParDE system from *Mycobacteria tuberculosis* [70,80]. We note that many studies rely on “over-expression” of toxin proteins to analyze the cellular impacts, resulting in non-native concentrations. However, effects can be observed at very low levels of toxin protein expression barely detectable by Western blotting, leading to the suggestion these could be reasonable estimates for the free toxin of an “activated” system [69]. Overall, it seems that gyrase-targeting TA systems may occupy a specialized niche that bridges protection at low-levels of expression and/or with attenuated toxicity, to a higher-level toxicity at prolonged exposure or concentration levels. Given that FicT toxins have also been identified as modulating both DNA gyrase and topoisomerase IV via adenylation [81], it remains to be determined if they will have similar impacts to heat tolerance or target protection.

Protection from thermal stress is noted for multiple members of RNA-degrading (“RNase”) type toxins, particularly those of the RelE-type fold. The YoeB toxin from *E. coli* protects thermal stress, and further, this is dependent on the proteases needed for antitoxin degradation, implying this protection arises from some direct action of the toxin [82]. Studies in the gram-positive bacteria *Streptococcus pneumonia* demonstrated genetic deletions of two *yoeB* loci and related RNase toxins were less hardy when exposed to oxidative conditions, while complemented mutants recapitulated wild type survival levels [83]. The structurally related GraT toxin from *Pseudomonads* similarly protects from temperature stresses but appears to function in response to lower rather than elevated temperatures [72]. Subsequent work with GraT highlighted the overall depression in metabolism in response to this toxin [84]. YafQ, again within the same structural class as the RelE/ParE family, was also found to be protective for bacterial growth at sub-optimal temperatures [85].

Other classes of toxin activities, particularly those that impact RNA lifetimes, have also proven to impart beneficial functions to host physiology. Three RelE toxins in the genome of *Mycobacterium tuberculosis* were identified as upregulated at the transcript level in response to altered oxygen levels or limiting nitrogen [86]. This study linked RNase toxin activation to proteome alteration by two-dimensional gel electrophoresis and mass spectrometry analysis of differential products. The RelBE TA system has been documented to be activated in response to nutritional stress [87], likely triggered in natural settings by high cell density [88] and similar to findings with *Acinetobacter baumannii* and *P. aeruginosa* HigBA systems [89,90]. These systems in *Mycobacterium tuberculosis* also provide protection to antibiotics but, importantly, did not induce persister cell formation [76]. It remains unclear if this is the “native” use for these; or, in other words, if the bacterial cells experience these conditions as part of their normal growth cycle and in turn utilize these toxins to slow growth and thus hedge survival. Further, considerable controversy still surrounds many earlier findings for specific details of the RNase toxin–starvation models [12,13,91].

Many of these studies rely on a reductive approach in defined growth conditions, whereas the use of TA systems within a natural ecological setting has been harder to access. The TA systems YefM-YoeB, Hha-YbaJ (Hha-TomB), and PasTI (also named RatA [92] to reflect its Ribosome Association toxin, or inhibition) harbored on the chromosome of extraintestinal pathogenic *E. coli* (ExPEC) strains were noted to promote increases in recoverable viable bacteria from the bladder and kidneys of a mouse model of infection [93]. The deletion of MazEF systems in *M. tuberculosis* reduced virulence in an animal model, as did Vap systems in *Haemophilus influenza* [94,95]. The Hha-TomB Type II system, which impacts translation through ribosomal interactions, has also been associated to increased *Salmonella typhimurium* survival in infection models [96]. Other studies have highlighted a role for TA systems in the survival of *Salmonella* within macrophages [97]. Subsequent studies have complicated this model and suggested that the effect resulted from slowed bacterial metabolism, which is a known impact from TA system activation [16,77]. Similar complexities that produce different outcomes have been highlighted for the MqsRA and MazEF systems in *E. coli* [98,99,100,101,102,103]. The impacts of chromosomal TA systems on their host cells are clearly affected by very specific interactions and by interwoven pathways. These have been well reviewed elsewhere, and are brought up here to illustrate the unsettled nature of these on-going studies and evolving conclusions [104,105,106,107].

The continued discovery of new TA systems highlights their broad capacity for integration into bacterial cells. The *Streptococcus pneumoniae* chromosomal PezAT system, a xenolog of the Epsilon-Zeta system [108], is located within an integrated pathogenicity island, and when deleted induced phenotypic changes that produced both beneficial (harder to lyse) and negative (more sensitive to cell wall antibiotics) effects for the cell [109]. The ParST, an mART-type that transfers an ADP-ribosyl group onto an enzyme involved in phosphoribosyl pyrophosphate synthetase, induces bacteriostasis when transplanted from its native *Sphingobium* host to *E. coli* [110]. This study notes the widespread distribution of the ParST system with enrichment in Proteobacterial classes. The diverse and highly integrated nature of TA systems and bacterial physiology is exemplified by the recently published work demonstrating some alarmone synthases are housed as TA loci [43]. These systems, named toxSAS, deviate from the traditional canon in encoding multiple antitoxins per synthase, or toxin, with both cognate and universal neutralization interactions [43]. It seems, then, that the diversity and functions of TA systems are expansive and may yet reveal new secrets of bacterial growth in the coming years.

### 2.2. Plasmid Selection and/or Addiction via Type II TA Systems

As mentioned above, chromosomal Type II TA systems were almost certainly acquired by invading genetic material. For example, a ParDE system in *P. aeruginosa* is located within the Pf1 prophage [66], and the widely-studied RelBE system in *E. coli* is located within a Qin prophage [63]. The *Vibrio cholera* superintegron on chromosome II, including an integrated and conjugative element (ICE) called SXT, is enriched with both antibiotic resistance cassettes as well as Type II TA systems [111,112]. *Neisseriaceae* and *Klebsiella* species have similar integrated genetic elements, including Type-IV Secretion System components connected to TA systems [47,113], as well as other polymorphic toxin systems resembling Type II TA systems [104,114,115,116]. The chromosomal localization of these TA systems coupled with demonstrated toxicity in the absence of the antitoxin strongly implies these likely functioned as an addiction system for loss of the genetic material, and as are mentioned here in the “addiction” category rather than above with other chromosomal TA systems (see Table 1).

The idea of plasmid addiction, also referred to as PSK, is generally found with low copy number plasmids and is predicated on the shorter half-life of the neutralizing antitoxin, allowing daughter cells to inherit portions of the parental cytoplasmic material including more stable toxins (Figure 1b) [57,117,118,119,120]. While this is also feasible for phage to utilize and thus mediate infections, as recently described for the Pf1 prophage in *P. aeruginosa* [121,122], this is more commonly used by Type III TA systems and therefore Table 1 focuses on well characterized examples of plasmid-based Type II systems. When the host bacterial cell lacks a corresponding mechanism to neutralize the inherited toxin proteins, a negative impact on growth is realized. However, some bacteria encode chromosomal antitoxins that can neutralize the plasmidic counterparts, referred to as “anti-addiction” (see Section 2.3, below).

Type II TA systems are widespread on plasmids where they mediate either addiction or plasmidic competition [8,12,123,124,125]. Further, because TA systems select for plasmid maintenance, they also then contribute to spread of AMR [8,11]. For example, the pUM505 plasmid (an IncI-type) from *P. aeruginosa* contains a pathogenicity island with an encoded *pumAB* TA system (a RelBE homolog) and numerous resistance genes, including a ciprofloxacin-modifying enzyme CrpC [126]. Strains containing this plasmid were more virulent in both *Caenorhabditis elegans* and mouse model infections. A recent analysis of plasmids carried in *Klebsiella* strains found a strong association of Type II TA systems and resistance genes, with enrichment for ParE, ParE-like, CcdB, and Vap-type PIN domain toxins within IncA/C- and IncH-type plasmids [47].

A recent preprint report nicely summarizes TA systems found on different Inc plasmids in *Enterobacteriaceae* [5]. For *Klebsiella pneumonia* strains, they can contain up to 11 different Inc types of plasmids, with approximately half of these being IncF-types [5]. These included 27 different Type-II families with *ccdAB* and *pemIK* the most common, consistent with previous reports on the pOXA-48 IncL/M-type plasmid [5,127] as well as previous work on *E. coli-*derived plasmids [128]. Interestingly, when compared to their counterparts in *E. coli,* the *ccdAB* loci show greater sequence divergence, while the *pemIK* systems are relatively well conserved [5]. Previous reports on IncX-type plasmids noted enrichment for RelE/ParE-type TA systems [123]. 

The literature presents at least one well-documented case of a CcdAB xenolog in *E. coli* O157:H7 that is neutralized by its plasmidic counterpart from the F plasmid, whereas the plasmid-borne toxin is only neutralized by the same (cognate) plasmidic antitoxin [73]. These homologous systems co-exist stably in the population, perhaps mediating a reverse addiction wherein the plasmid is retained specifically to neutralize the chromosomally-integrated copy of the toxin. What is clear is that this type of addiction requires an antitoxin protein to interact with multiple different toxins, and that this standard PSK “addiction” model would not typically work if chromosomal antitoxin xenologs could cross-interact with plasmid-derived toxins, thus requiring a type of directional insulation for cross-interactions.

### 2.3. Type II TA Systems Mediating Anti-Addition through Antitoxin Cross-Interactions

An alternative outcome to the Addiction model discussed in Section 2.2 is Anti-addiction, wherein chromosomally encoded TA systems protect the host bacteria against PSK mediated by their plasmid-encoded counterparts through cross-interactions (Figure 1c) [7,59,63]. Recent work highlights an analogous model for Tn3 transposons [4] and prophage sequences [64]. Other experiments have highlighted that PSK is not necessarily the driving force for retention of TA systems, and instead it is due to plasmid competition, such that a TA system with homologs carried on two plasmids would “compete” for cross-toxin neutralization, leading to “survival” or retention of the “winning” plasmid [147]. Additional support comes from observations that PSK does not actually result in complete sterilization of the culture, but rather a decreased viability that recovers over time [148].

Anti-addiction was clearly demonstrated for the *Erwinia chrysanthemi* chromosomal antitoxin CcdA, which interacts with and neutralizes the incoming F1 plasmidic CcdB toxin [59]. A similar modality is mediated by the phage-derived protein Dmd, which serves as an antitoxin for the RnlA and LsoA toxins in *E. coli* [149]. Anti-addiction is closely linked to “abortive infection” of phages, which center more on the Type IV type of TA system including ToxIN and AbiEI [31,35,150,151,152]. The presence of orphan antitoxins that appear to encode a protein, such as used in Type II TA systems, readily supports the anti-addiction model, but this remains to be demonstrated as a common usage of these orphans [13]. 

This model of TA system functions is dependent on the ability of antitoxin proteins to cross-react, such that they can neutralize the toxin on invading genetic material. Given the dogma of cognate toxin–antitoxin interactions, the current review will revisit examples of known non-cognate interactions (with some previously reviewed in [28]) as well as the feasibility of this occurring in selected systems.

## 3. Conservation of Type II TA System Folds and Cognate Antitoxin Interactions 

Numerous experimentally determined structures are available for Type II TA systems, and these confirm that although sequence conservation is low, their structures are highly conserved and can be used to group them into superfamilies [28,51,153]. These classifications highlight the modular nature of toxin family interactions with different antitoxin families, consistent with propagation by horizontal gene transfer [28,154]. Antitoxins are the critical regulatory component for Type II systems, wherein they (typically) contain an N-terminal DNA binding motif used for autoregulation of the TA operon, and a less structured C-terminal domain to neutralize the toxin’s activity. Selected systems that have the antitoxin domains reversed, such that the N-terminal region mediates toxin neutralization [89,155,156,157,158,159], while some antitoxins are limited to only the toxin-binding domain [160]. Antitoxins neutralize toxins by either by blocking the active site or by causing conformation changes that prevent toxin interaction with the cellular target [51,154,161]. 

### 3.1. Toxin Families Share Conserved Folds but Interact with Different Families of Antitoxins

**Figure 2:**
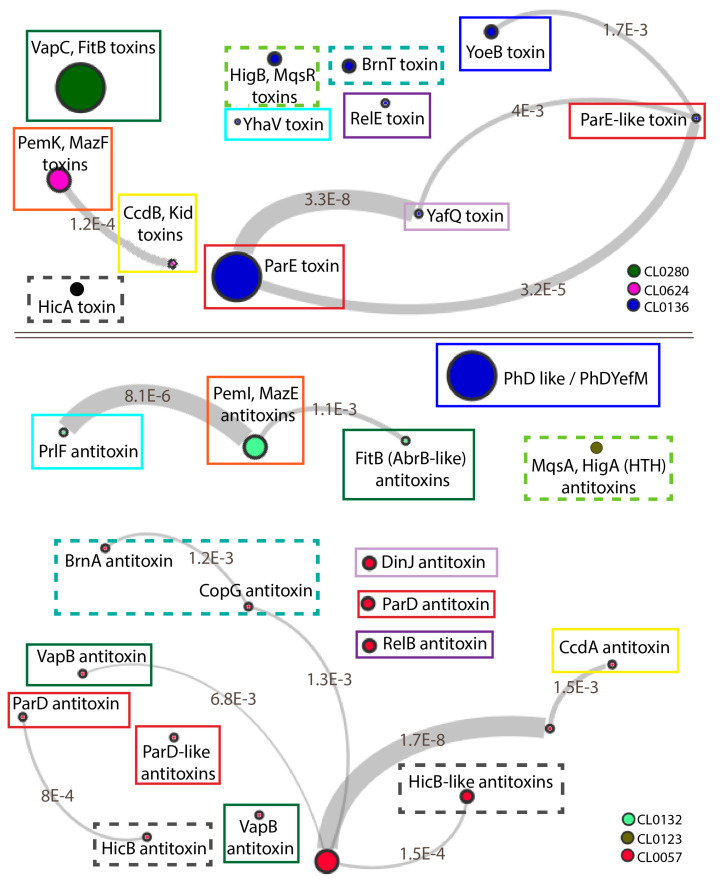
TA families can be grouped into Pfam “clans” (here connected by grey lines) based on Hidden Markov models, although toxin families can interact with antitoxin families from disparate clans. The force-directed diagram indicates the families that toxins and antitoxins belong to providing a predictive effect for noncognate toxin-antitoxin interaction. Patterns are adapted from the Pfam database, where the size of the circle corresponds to the number of sequences within a given family while the color corresponds to the clan. Colored boxes (dashed or solid lines) indicate cognate toxin (top) and antitoxin (bottom) pairs. The linkage between each circle indicates the E value between related families, with a lower limit of 10^−3^ to be considered significant.

The Pfam database utilizes Hidden Markov-models to associate similar protein families exhibiting a conserved fold or annotated function into “clans” [162]. When viewed for Type II TA systems, there are three large mostly toxin-containing clans, in addition to three common antitoxin-containing clans (Figure 2). A key feature of these is the mix-and-match nature of different antitoxin families with toxins from the same family, and vice versa. The toxin proteins are categorized into a PIN-type clan, encompassing Vap and Fit family toxins, a RelE/ParE-type clan, and a CcdB/PemK-type clan. Among these, the PIN-type VapBC systems and the RelBE-like systems appear to be the two largest families [49]. Some toxins are self-contained families that do not correlate into a larger clan (at least to date), including those involved in post-translation modifications such as the HEPN, FIC, and GNAT types (for these, see other recent reviews [7,12,27]). Previous work demonstrated the strong conservation of DNA binding motifs, generally a helix-turn-helix (HTH, see Figure 2) or ribbon-helix-helix (RHH, see “CL0057”, Figure 2), in addition to the Abr-like DNA binding domain for different antitoxin families (Figure 2) [28,154,163]. 

**Figure 3:**
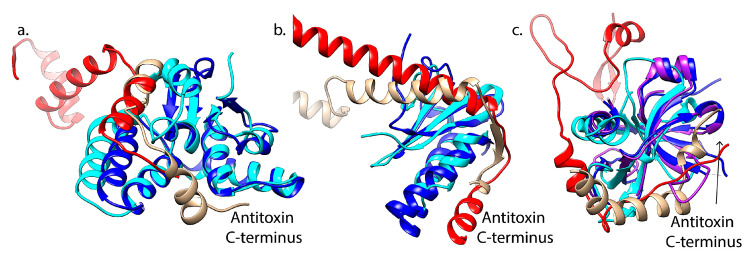
Shared toxin structures are neutralized by distinct antitoxin family structures, with variation evident in the C-terminal ends of the antitoxins. (**a**) Superposition of VapC (cyan) and FitB (blue) toxins reveals an overlap in interaction surfaces with antitoxin VapB (tan) and FitA (red). (PDB IDs 3DBO [167] and 2H1O [169]) (**b**) Superposition of RelE (cyan) and ParE (blue) toxins reveals an overlap in interaction surfaces with antitoxin RelB (tan) and ParD (red). (PDB IDs 4FXE [176] and 3KXE [177]) (**c**) Superposition of Kis toxin (light blue) with MazF (cyan) and CcdB (blue) toxins reveals an overlap in interaction surfaces with antitoxin MazE (tan) and CcdA (red). (PDB IDs 1M1F [178], 1UB4 [179], and 3HPW [180]).

The CL0280 group of toxins contains families that interact with either CL0132 or CL0057, depending on the toxin family. The toxins in this family all share a PIN domain that forms a compact RNA-binding with three highly conserved acidic residues required for metal-dependent endonuclease activity [153,164,165,166]. Within this fold, the VapC-type toxins found in *M. tuberculosis*, *Shigella flexneri* and *Rickettsia felis* have minor variations in numbers of specific secondary structure elements [167,168]. This fold also encompasses the FitB toxin family [169]. While the VapC and FitB toxins share structural homology, their cognate antitoxins, VapB and FitA, are located in different Pfam clans. These mediate similar yet distinct interactions their cognate toxins, with distinct structures at the more N-terminal part of FitA as compared to the C-terminus of VapB (red versus tan ribbons, Figure 3a). The VapB antitoxin is part of the RHH antitoxin family (CL0057), while the FitA antitoxins more closely correlate with the Mnt-like repressors (CL0132). Within this family, the conserved toxin fold thus interacts with antitoxins from two different structural Pfam “clans” [168,169,170].

Overall, toxins segregated in the CL0136 group are paired with distinct antitoxin families in the CL0057 group with a few notable exceptions. This toxin group is comprised of the RelE/ParE family of toxins containing a shared microbial RNase fold but variability in the specific active site amino acids as well as extensions at the C-terminus [13,171,172]. While most of the toxin members mediate RNA cleavage, as recently reviewed [13], the ParE toxins are unique in inhibiting DNA gyrase through an as yet unknown mechanism [71,80,173]. Despite the shared fold and mechanism, subfamilies appear to exist with both ParE-types and RelE-types, including a wide range of RelE-like RNases including HigB, YoeB, YafQ, BrnT, and MqsR (Figure 2) [27,171,172,174]. This family also includes an integrated phage-derived tripartite ParE system that appears to not mediate RNA cleavage or DNA gyrase inhibition [160,175]. Interactions with antitoxins span five different specific families in CL0057, as well as antitoxin members of CL0136 and CL0123 (Figure 2) [1,51,174]. These interactions are mediated by analogous surfaces among these distinct families, yet with distinct sequence differences and, in particular, the ParD antitoxins have longer C-terminal regions versus the RelB-type antitoxins (Figure 3b).

The CL0624 toxin group similarly interacts with antitoxins in either CL0132 or CL0057. This Pfam “clan” of toxins includes the CcdAB, Kis-Kid, PemIK, and MazEF Type II TA systems. CcdB toxins act by inhibiting DNA gyrase, although using distinct mechanisms as the ParE-type toxins [181,182,183]. CcdB toxins have a striking structural similarity with the toxins Kid and PemK, which are endoribonuclease encoded by the Kis-Kid (*parD*) TA system found on the R1 plasmid and the PemIK system found on the R100 plasmid [133,134,135,184,185]. This structural family is further expanded by PemIK xenologs in *Bacillus anthracis* [186] and *E. coli* (named ChpAB) [187]. The MazF toxin is a structurally homologous RNase, although its interaction with the antitoxin is distinct among this clan (Figure 2) [179]. The MazE antitoxin consisting of a looped–hinge–helix (LHH) fold, the N-terminal region of Kis antitoxin has a unique LHH fold [188], while the CcdA antitoxin has an RHH fold [189]. It is clear that cross-interactions with non-cognate toxins in theory could occur, as the antitoxins are classified based on the DNA binding domain rather than the toxin-binding domain, and the toxin surfaces complexed with antitoxin are largely overlapping (Figure 3c). 

### 3.2. Interactions with Cognate Antitoxins

For type II TA systems, the neutralization between the toxin and the antitoxin mediate direct protein-protein interaction. Under normal condition, the antitoxin neutralizes its cognate toxin as well as its expression to prevent its toxicity, whereas under environmental stressors, the antitoxins are believed to follow a common proteolytic degradation to release toxins, allowing it to kill cells or return cells to a dormant state [12,154,163,190]. The insulation within homologous systems has been well documented [190,191,192], such that the direct protein-protein interactions of toxins and antitoxins, the central mechanism of toxin control for Type II TA systems, appear to be highly specific [175,193,194].

This is exemplified within *Caulobacter crescentus,* which contains four chromosomal RelBE systems in addition to three functional ParDE systems; while these toxin families have structural similarity, they are neutralized by different classes of antitoxins [70,154]. Using a deletion approach, each cognate pair was demonstrated to have no cross-reactivity by virtue of a lack of survival when cognate antitoxins were deleted [70]. Similarly, seventeen TA systems in *V. cholera* were demonstrated to have no cross-reactivity of antitoxins [195]. Further, the relatively unique tripartite systems paaA-ParE found in integrated prophage regions in *E. coli* O157:H7 also do not cross-react [175]. 

*M. tuberculosis* encodes up to 55 different cognate VapBC systems, which were the subject of a recent study that, guided by available crystal structures, made predictions on the amino acids in the interface of each cognate pair [196]. They were able to identify sub-clusters within both toxins and antitoxins that were more likely to contain cross-interacting pairs. These predictions corroborate previous work that experimentally demonstrated high insulation thus limited crosstalk between these systems in different Vap sub-clusters [197]. Within a sub-cluster, however, possibility for cross-talk increases [196], and has previously been predicated on the identity of the C-terminal (30 amino acids) of at least a few of these VapB antitoxins [198] 

## 4. Feasibility for Cross-Interactions of Type II TA Systems

A recent review provided a concise view of the models for TA system functions [12], and we will attempt to not repeat those here. The Anti-Addiction/Plasmid Competition model presents an attractive explanation for the long-sought functional significance of chromosomal TA systems. Based on this, we can draw the following suppositions that should be fulfilled for this model: That the chromosomal TA system will have a “match” on some invading genetic material (transposons, phage, integrons, and plasmids), and, that the chromosomal antitoxin will match the invading toxin well enough to neutralize it.

Instead, our intent is to more closely examine some of these ideas from the structural and molecular standpoint, and in particular, is predicated on the feasibility of antitoxin cross-reactions required to fulfill prevailing models of TA system addiction and anti-addiction functions.

### 4.1. Examples of Antitoxin Cross-Reactivity

Many TA systems, despite having low sequence similarity, exhibit a similar folding structure [25,153,154,199]. Typically these structures enforce interactions limited to cognate partners, providing insulation from cross-reactivity. However, some examples of cross-interactions have been noted to occur both between chromosomal and plasmid-borne TA systems. 

Such complex cross-regulations were observed between three different *M. tuberculosis* RelBE-like modules, RelBE (Rv1246c-Rv1247c), RelFG (Rv2865-Rv2866) and RelJK (Rv3357-Rv3358). Using in vitro and cell survival assays, it was demonstrated that the RelB antitoxin can cross-neutralize the non-cognate RelG toxin, but RelB can also enhance the toxicity of the RelK toxin in cell survival assays, although the molecular basis for this remains unclear. On the other hand, RelF, the antitoxin of RelG, is able to enhance the toxicity of RelE which causes severe inhibition on bacterial growth compared to the set only expressing RelE [191]. A similar form of cross-interaction was noted for two RelB proteins encoded in the *Y. pestis* CO92 genome, although these only differ by three amino acids [200]. Cross-reactivity has been documented for the CcdAB system carried on the F plasmid [59,73]. The *Erwinia chrysanthemi* chromosomal CcdA antitoxin interacts and neutralizes the incoming F1 CcdB toxin that would otherwise kill the cell [59]. Subsequent studies noted that amino acid changes Asn 69 to Tyr in the chromosomal CcdA antitoxin and the plasmid-derived Tyr 8 Arg in the CcdB toxin affects cross-interactions, resulting in weak plasmid-derived toxin binding to chromosomal antitoxin [68,184].

Interestingly, the Kid and MazE antitoxins are able to mutually interact and partially neutralize the toxicity mediated by the non-cognate family [188]. Similarly, the CcdA and Kis antitoxins are able to cross-interact with non-cognate toxins from non-cognate family members (Kid and CcdB, respectively) [176]. CcdA binding enhances the endoribonuclease activity of Kid by triggering a conformational change that promotes interaction with its target RNA, while the Kis antitoxin effectively neutralizes the toxicity of the CcdB toxin [184]. The different effect of antitoxins on the non-cognate toxins results from their overlapping yet distinct binding sites along the toxin (Figure 3c), and as well as from potential differences in their DNA binding regions [184,188,189].

The VapBC systems in *M. tuberculosis* are highly specific for their cognate pairs; a given VapB antitoxin is not able to neutralize a non-cognate VapC toxin [190]. This was also demonstrated for different ParDE families that interact in a highly specific manner [161]. However, both studies identify the determinants for antitoxin recognition as well as the specificity and insulation of crosstalk between different TA systems. Mutating a single tryptophan amino acid in the VapB1 antitoxin from non-typeable *Haemophilus influenza* renders it to antagonize both its cognate VapC1 toxin and its non-cognate VapC2 toxin [190]. In the ParDE family, switching antitoxin residues 60, 61 and 64 in ParD3 is sufficient to alter the specificity from the cognate ParE3 to non-cognate ParE2 [161]. Those results suggested the possibility of breakage to the insulation of crosstalk and specificity between different TA systems. 

Other experiments were able to generate lab-derived cross-reaction of antitoxins. Chromosomal antitoxins MazF and ChpB were mutated and constructs were selected for their ability to then neutralize plasmid-derived PemK toxin located on R100/R1 plasmids [201]. Similarly, using chemical mutagenesis of the ChpBI system that then selected for cross-neutralization against the Kis toxin and noted this mutated version could also still neutralize the ChpK toxin [201]. Chromosomal MazE is a homolog of Kis on the R1 plasmid, and can neutralize the plasmid-derived Kid although the interaction is weaker than with the cognate pair. [188,202] *M. tuberculosis* encodes seven annotated MazEF systems and two additional MazE antitoxin homologs of the *E. coli* systems, although these are not paired with the normal cognate MazF toxin [203]. They identified a “network” of non-cognate interactions, such that one of the tested MazE antitoxins interacts with two different MazF toxins. Another set of non-cognate interactions is completely reciprocated, such that a MazE antitoxin can interact with non-cognate VapC type toxins, and their cognate VapB antitoxins interact with the cognate MazF toxin as well as the non-cognate VapC toxin [203].

### 4.2. Orphan Antitoxins

Given the widespread nature of TA systems, it is not surprising that many partial systems are annotated. However, antitoxins that lack a cognate toxin may be particularly important for Anti-Addiction functions, as they can provide a source of toxin neutralization independent of any inherent addiction properties themselves. Orphan antitoxins have been noted in bioinformatics studies of bacterial genomes, including pathogens associated with high incidences of antibacterial resistance. The *Bartonella schoenbuchensis* type-IV secretion system is encoded on a conjugative plasmid, pVbh, where it encodes fourteen canonical TA systems as well as four orphan antitoxins [104]. While the TA systems likely mediate PSK, which is an addiction function, the orphan antitoxins are inferred to likely function as anti-addiction modules.

A recent study analyzed the genome sequences of 259 species of *Klebsiella pneumonia* complex strains and was able to predict up to 2253 orphan antitoxins [47]. These were proposed to encode remnants of degraded TA systems, or to be regulators of other (unidentified) TA pairs, or as anti-addiction modules to prevent foreign genetic material from being retained in the cell [47]. The sequences surrounding these orphan antitoxins were screened for similarities to known TA system arrangements, which revealed a high percentage were likely to be degraded from previously intact canonical systems. However, around 20% of those identified appeared to be genuine orphan genes, and further, around half of these open reading frames encoded a protein with canonical features of antitoxins [47]. 

A similar study mined *Mycobacterium tuberculosis* (Mt) genome sequences and identified both VapB orphan antitoxins and VapC orphan toxins [196]. The orphan toxins were more closely related to other paralogous toxins, whereas the orphan antitoxins were not similar to other known antitoxins. *Acinetobacter baumannii* also encodes numerous orphan antitoxins, as well as pairings of canonical antitoxin or toxins with non-TA system proteins [145]. A subset of the Mt orphan toxins is closely related to TA systems in *Mycobacterium marinum,* implying horizontal gene transfer between the two organisms [196]. However, variations in the active site residue raised questions about the potential for these orphan toxins to remain active [196]; however, it is not clear if these amino acid changes would impact interactions with antitoxins. Similarly, a *parDE* loci in *Caulobacter crescentus* encodes a *parE* pseudogene, resulting in a ParD antitoxin with no apparent mate, technically an “orphan” [70]. A TA system found in *Shigella flexneri* encodes a non-functional toxin, YacB, due to a frameshift mutation causing a premature stop codon [146]. The cognate antitoxin, Orf176, has previously been identified as the YacA antitoxin paired with a YacB toxin on *E. coli* plasmid pWR100 [146]. While it can be considered an “orphan” antitoxin, interestingly it is one of three such orphans that were validated substrates for type-III secretion in *Shigella* [204].

### 4.3. Predictions of Antitoxin Cross-Reactivity

The idea of cross-interactions in anti-addiction (Figure 1c) is predicated on the ability of one partner to interact with multiple others; the likelihood of this increases as structures or sequences are more conserved. We undertook an examination of 14 available structures, identified using tools at the Protein Databank interface [205,206], for chromosomal RelE-type toxin interactions with their corresponding antitoxins to identify sequence conservation that may indicate the ability for cross-reactions [154,159,174,176,207,208,209,210,211,212,213,214,215,216]. However, we note that within this set, the RelBE complex from *E. coli* is represented by two structures (PDB IDs 2KC8 and 4FXE, [176,207], respectively), as is the Doc toxin from the P1 bacteriophage (PDB IDs 3DD7 and 3KH2, [154,210], respectively), limiting the structure set to twelve unique complexes. The overall secondary structure and interaction of cognate RelB-like antitoxins are conserved, with two α-helices separated by a β strand and wherein this β strand typically pairs with a strand from the toxin to form an extended cross-molecule β-sheet. 

**Figure 4:**
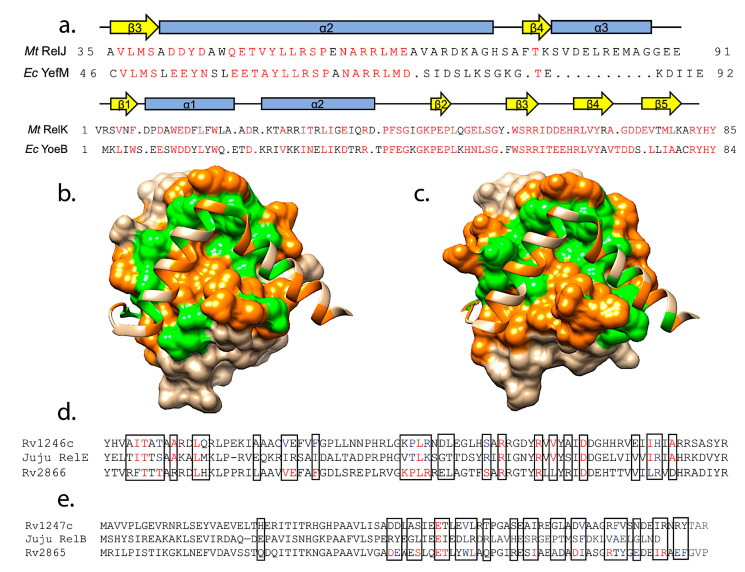
Potential cross-interactions of RelB/YefM antitoxins identified from analysis of sequence and structural conservation among available experimentally determined structures. (**a**) Sequence alignments (top, antitoxins; bottom, toxins) depict conserved amino acids (red), with secondary structure indicated above. (**b**) Surface of *Mycobacteria tuberculosis* toxin RelK with ribbon backbone of *M. tuberculosis* antitoxin RelJ (C-terminus is to the right); coloring is orange for hydrophobic interaction points, and green for polar interaction points. (PDB ID 3OEI, [212]). (**c**) Surface of *E. coli* toxin YoeB with ribbon backbone of *E. coli* antitoxin YefM; coloring as in b. (PDB ID 2A6Q, [211]). (**d**) Sequence alignment for Rv1246c, a RelE toxin found in the chromosome of *M. tuberculosis* and exhibiting similarity to a phage-derived sequences. This is compared to Rv2866, another *M. tuberculosis* RelE toxin with an available crystal structure (PDB ID 3G50, [212]), which was used to delineate likely protein–protein contacts for the RelE toxin and RelB antitoxin (black boxes). Red text indicates conservation, while blue indicates similarity. Note, Rv1246c is colored relative to the phage derived sequence. (**e**) The same type of sequence analysis was carried out for the respective RelB antitoxin sequences (colored and labeled as in d).

We utilized the PISA webserver [217] to list the interacting amino acids, visualized these with UCSF Chimera [218], and then inspected the different interactions by eye. Within these structures we identified two sets of antitoxins that have very similar sequences at the toxin binding sites (Figure 4a). In particular, the RelJ antitoxin from *M. tuberculosis* and the *E. coli* YefM antitoxin have high sequence similarity at the region of interaction with toxins (residues 39–77 of RelJ and residues 51–89 of *E. coli* YefM) at 56.4%(or 22 out of 39 amino acids). Further, the distribution of polar versus hydrophobic amino acids at the antitoxin–toxin interfaces are also highly correlated (Figure 4b,c). We also identified that the sequences of *E. coli* HigA and *S. flexneri* HigA are identical throughout the toxin interactions regions. Overall, the complexes adopt the same structure with some minor differences in toxin loops visualized in the crystal structures (PDB IDs 4FXE and 2KC8, [159,216]). Not surprisingly, the HigB toxins from these two organisms are identical [159,216]. These examples lend some support to the idea that a structure-based approach should be able to predict cross-interacting pairs.

With a similar objective in mind, we undertook a search of phage-derived sequences to assess any potential cross-reactive TA systems as compared to *M. tuberculosis,* such as would be expected for an anti-addiction function of chromosomal systems (Figure 1). The toxin and antitoxin sequences for *M. tuberculosis* (Mtb) strain H37Rv were obtained from the toxin-antitoxin database and included the RelBE, HigBA, and VapBC families [49]. The sequences were used as a query to search the Actinobacteriophage (previously “Mycobacteriophage”) Database that contains approximately 3400 sequenced phage, of which 1900 are known to originate from Mycobacterial hosts (https://phagesdb.org; [219]). The resulting BlastP resulting sequences were surprisingly poorly matched; as such, any with an E-value of 0.10 or less were aligned with the H37Rv toxin or antitoxin to which a similarity was indicated [176,213,220,221]. 

Among the antitoxin sequences, Mtb VapB8, VapB13, and VapB40 all had a potential match with the phage database; however, each had only one conserved amino acid likely to be in the interface. Two HigA antitoxins (Rv2021c and Rv3183) presented sequence matches in the E 10^−3^ to E 10^−4^ range, and similarities were limited to approximately 30 amino acids at the toxin-binding interface. For Rv3183, also known as HigA3, 18 of 31 amino acids present an exact match while an addition four are conserved; further, this antitoxin sequence is derived from a *Mycobacterium-*derived phage. However, this appears to be an orphan antitoxin, as the annotated open reading frames on either side of this gene do not contain similarity to the HigB (or any other) toxin. *M. tuberculosis* HigA2, annotated as Rc2021c, matches a sequence derived from a phage originating in Propionibacterium. Similar to the HigA3 antitoxin, this HigA2 antitoxin contains 22 identical and 12 similar amino acids out of 53 total, although these matches are more central to the protein so would be expected to span the toxin binding domain and the DNA binding domain. While it contains a reasonably-sized open reading frame in the toxin position (150 amino acids), the encoded protein failed to match any known sequences in the TADB or any named protein in a Blast search at the NCBI (all were “hypothetical”), indicating perhaps this is a novel toxin or an orphan HigA antitoxin embedded within a different genetic context.

Three VapC toxins (VapC5, VapC28, and VapC42) had an E-value of 0.10 or less with the matching phage sequences. The most successful homologs were to an experimentally verified RelE toxin (Rv1246c) with an E-value to the best-matched sequence of 2 E 10^−5^. This sequence originated from the “Juju” phage, with 33% identity and 50% conservation, as well as strong matches with 33 other phage entries [191,222]. Among these phage, the top twelve matches originated from a Gordonia host, while the remainder are from *Mycobacterium smegmatis*. Each of these phage (where genomes were annotated) contains an open reading frame for a RelB antitoxin just preceding the RelE-type toxin. 

These sequences were analyzed for conservation of interaction sites based on the closest matching RelBE complex, the RelBE2 system (Rv2865-2866, PDB ID 3G5O, [212]). Assuming a conservation of interaction sites between RelE toxin and RelB antitoxin, at these sites of interaction the Rv1246c RelE toxin and the Juju phage RelE toxin have the same amino acids at 11 positions, conserved amino acids at 7 positions, and different amino acids at the remaining 9 positions (Figure 4d). When comparing the same chromosomal RelE toxin to its closest match in the PDB (Rv2866) there are 15 identical amino acids, 3 conserved, and 9 different amino acids at the interface (PDB ID 3G5O, [212]). 

Given that the match was based on toxin sequences, we speculated that the corresponding RelB antitoxin sequences would be similar. The same type of analysis revealed limited conservation of antitoxins, with identical amino acids at one position, conserved amino acids at only 3 positions, and different amino acids at the remaining 22 positions (Figure 4e). As a marker of comparison, when the chromosomal Rv1246c antitoxin was compared to the crystal structure of Rv2865 there are 10 identical amino acids, 8 conserved, and 8 different amino acids at the toxin-contacting interface (PDB ID 3G5O, [212]). 

This leaves an open question of—do toxins with similar antitoxin-interaction amino acids cross-react with disparate antitoxin sequences? Or, another way to phrase this might be, could the *M. tuberculosis* chromosomal RelB antitoxin (Rv1247c) neutralize an invading phage RelE toxin, and in so doing mediate anti-addiction? Further, the recovery of the phage from related Gordonia bacterial species could indicate an anti-addiction function when this chromosomal matching RelE toxin is present, or, simply could represent a sampling bias for environmental phage collection comprising the sequence database [219].

## 5. Discussion

TA systems are abundant on bacterial chromosomes with a seemingly high insulation from non-homologous interactions. To date, such cross-interactions have been detected when changes to either partner accumulate. Given that the premise of anti-addition offered by chromosomal systems towards mobile genetic elements requires cross-reactivity, it seems likely that it would be present in some integrated chromosomal systems as well. However, only limited cross-reactions have been observed between chromosomal and mobile genetic elements systems, perhaps due to bias in the type of experiments and functions of TA systems pursued. 

It is not possible to reliably predict cross-interactions based on the currently available molecular characterizations, particularly if only a few amino acid changes are required in either partner. It may be that the natural variation that leads to such cross-reactivity does not follow such a minimalistic approach, with instead many more mutations providing a cumulative basis for an anti-addiction paradigm. It is clear that a more comprehensive understanding and screening for cross-interactions of non-cognate TA system partners would provide welcome insights into the functional possibilities for these intriguing systems.

## Figures and Tables

**Table 1:** Plasmidic TA systems characterized in the literature.

TA System	Plasmid	Host Bacteria	Chromosomal Homolog/Xenolog	Citations
ParDE	RK2 */RP4 *	*(Enterobacteriaceae) Escherichia coli*	ParDE	[55,79,129]
RelBE	P307, IncB/C	*E. coli*	RelBE	[130,131]
VapBC	IncF	*E. coli*		[131]
StbDE	R485	*Morganella morganii*		[132]
CcdAB, Kid/Kis (PemKI)	F, R1/R100, pCHP91	*E. coli, Erwinia chrysanthemi, Staphylococcus*	MazEF	[118,133,134,135,136]
PhD-Doc	Bacteriophage P1	*E. coli*		[137]
HigBA	pRTS1	*Proteus vulgaris*		[138]
Tad-ata	pAMI2	*Paracoccus aminophilus*		[139]
Axe-txe	pRUM	*Enterococcus faecalis*	YoeB-YefM	[140,141]
TasA-TasB	pGI1	*Bacillus thuringiensis*		[125]
VapBC	pMYSH6000	*Shigella flexneri*		[142]
AtaRT	pB171-like plasmid	*E.coli*	AtaRT	[143]
Omega-epsilon-zeta	pSM19035 *, pVER1/2	*Streptococcus pyogenes. Enterococcus faecium*	PezAT	[141,144]
RelBE, HigBA, HTH/GNAT, SplTA	p3ABAYE	*Acinetobacter baumannii*		[145]
YacAB	PWR100	*E. coli*		[146]

* Indicates a broad-host range plasmid.
